# Limited protection of pneumococcal vaccines against emergent *Streptococcus pneumoniae* serotype 14/ST876 strains

**DOI:** 10.1007/s15010-023-02110-y

**Published:** 2023-11-02

**Authors:** Yinle Lan, Lin Liu, Dongping Hu, Lihong Ge, Xi Xiang, Minfei Peng, Ying Fu, Yanfei Wang, Shuxian Li, Yan Chen, Yan Jiang, Yuexing Tu, Jorge E. Vidal, Yunsong Yu, Zhimin Chen, Xueqing Wu

**Affiliations:** 1 Department of Pulmonology, Children’s Hospital, Zhejiang University School of Medicine, National Clinical Research Center for Child Health, Hangzhou, Zhejiang China; 2 Laboratory Medicine Center, Department of Clinical Laboratory, Zhejiang Provincial People;s Hospital, Affiliated People’s Hospital, Hangzhou Medical College, Hangzhou, Zhejiang China; 3 Department of Infectious Disease, Affiliated Dongyang Hospital of Wenzhou Medical University, Dongyang, Zhejiang China; 4 Department of Clinical Laboratory, Children’s Hospital, Zhejiang University School of Medicine, National Clinical Research Center for Child Health, Hangzhou, Zhejiang China; 5 Department of Clinical Laboratory, Affiliated Jinhua Hospital, Zhejiang University School of Medicine, Jinhua, Zhejiang China; 6 Department of Clinical Laboratory, Taizhou Hospital of Zhejiang Province affiliated to Wenzhou Medical University, Taizhou, Zhejiang China; 7 Department of Clinical Laboratory, Sir Run Run Shaw Hospital, Zhejiang University School of Medicine, Hangzhou, Zhejiang China; 8 Key Laboratory of Precision Medicine in Diagnosis and Monitoring Research of Zhejiang Province, Hangzhou, Zhejiang China; 9 Department of Infectious Diseases, Sir Run Run Shaw Hospital, Zhejiang University School of Medicine, Hangzhou, Zhejiang China; 10 Key Laboratory of Microbial Technology and Bioinformatics of Zhejiang Province, Hangzhou, Zhejiang China; 11 Regional Medical Center for National Institute of Respiratory Diseases, Sir Run Run Shaw Hospital, Zhejiang University School of Medicine, Hangzhou, Zhejiang China; 12 Department of Critical Care Medicine, Tongde Hospital of Zhejiang Province, Hangzhou, Zhejiang China; 13 Department of Cell and Molecular Biology, Center for Immunology and Microbial Research, University of Mississippi Medical Center, Jackson, MS USA

**Keywords:** *Streptococcus pneumoniae*, One-child policy, ST876, Serotype 14, Vaccine escape

## Abstract

**Purpose:**

*Streptococcus pneumoniae* (Spn) is a major cause of child death. We investigated the epidemiology of *S. pneumoniae* in a pediatric fever clinic and explored the genomics basis of the limited vaccine response of serotype 14 strains worldwide.

**Methods:**

Febrile disease and pneumonia were diagnosed following criteria from the WHO at the end of 2019 at a tertiary children’s hospital. Spn was isolated by culture from nasopharyngeal (NP) swabs. The density was determined by *lytA*-base qPCR. Isolates were serotyped by Quellung and underwent antimicrobial susceptibility testing. Whole-genome sequencing was employed for molecular serotyping, MLST, antibiotic gene determination, SNP calling, recombination prediction, and phylogenetic analysis.

**Results:**

The presence of pneumococcus in the nasopharynx (87.5%, 7/8, *p* = 0.0227) and a high carriage (100%, 7/7, *p* = 0.0123) were significantly associated with pneumonia development. Living with siblings (73.7%, 14/19, *p* = 0.0125) and non-vaccination (56.0%, 28/50, *p* = 0.0377) contributed significantly to the Spn carriage. Serotype 14 was the most prevalent strain (16.67%, 5/30). The genome analysis of 1497 serotype 14 strains indicated S14/ST876 strains were only prevalent in China, presented limited vaccine responses with higher recombination activities within its *cps* locus, and unique variation patterns in the genes *wzg* and *lrp*.

**Conclusion:**

With the lifting of the one-child policy, it will be crucial for families with multiple children to get PCV vaccinations in China. Due to the highly variant *cps* locus and distinctive variation patterns in capsule shedding and binding proteins genes, the prevalent S14/ST876 strains have shown poor response to current vaccines. It is necessary to continue monitoring the molecular epidemiology of this vaccine escape clone.

**Supplementary Information:**

The online version contains supplementary material available at 10.1007/s15010-023-02110-y.

## Introduction

*Streptococcus pneumoniae* is a Gram-positive pathogen that causes various pneumococcal diseases (PDs), in which pneumonia is an infection of the lower respiratory tract mainly affecting young children and the aged [1, 2]. The typical symptoms of pneumonia include cough, tachypnea, and fever, which make the pediatric fever clinic one of the first options for Chinese children to be diagnosed with the etiology and undergo appropriate management of pneumococcal disease [3]. Studying the epidemiology of pneumococcal pneumonia in Chinese pediatric fever clinics, therefore, can be a critical surrogate for gathering epidemiological information, leading to effective disease prevention campaigns and successful management of PD.

A well-known risk factor for developing pneumonia is the colonization of pneumococcal strains in the upper respiratory tract, known as carriage. Several studies have demonstrated that pneumococcal carriage is more prevalent in children living with siblings [4–6]. China recently lifted the one-child policy; therefore, it is timely to evaluate the potential risk in this country of an increased burden of pneumococcal disease due to families now having more than one child in the household. In support of this, most reports of the burden of pneumococcal infections in China have been conducted retrospectively, using pneumococcal strains collected during the one-child policy era, spanning ~ 36 years.

Vaccination with pneumococcal conjugate vaccines (PCVs) has proven effective in preventing invasive pneumococcal diseases (IPDs), which pose a significant threat to children’s health [7, 8]. However, PCVs are not currently included in the Chinese national immunization program for young children, resulting in low PCV coverage in China, and have had little impact on the burden of pneumococcal infections [9, 10]. For example, PCV coverage was reported still below 30% at the end of 2019 in Zhejiang province, where the current study was conducted [11]. The most prevalent strains belong to vaccine types 19F, 19A, 6B, 6A, 23F, and 14, with serotype (S)14 being the most predominant strain causing invasive pneumococcal diseases (IPDs) [10, 12, 13]. China has observed a significant shift over time in the genetic diversity of S14 strains, including shifting from CC875, prevalent worldwide, to CC876, which is only prevalent in China, and a significant increase in resistance to beta-lactam antibiotics [14]. In other countries, S14 strains were predominant worldwide before the introduction of PCVs, but their prevalence has since decreased after the national implementation of pneumococcal vaccines [15]. However, the genetic makeup of S14 strains varies across different regions. Given that S14/ST876 is only prevalent in China, we have a unique opportunity to investigate the molecular and genetic basis of this new S14 clone and compare it with S14 strains isolated worldwide.

Thus, in the current study, we collected NP swabs from children under 5 admitted to the pediatric fever clinic in 2019, 3 years after the one-child policy was lifted. We conducted microbiological and genomic analyses of pneumococcal strains to understand the characteristics of carriage strains and strains causing PD. Because we isolated a number of S14 strains that escaped vaccination, we performed comprehensive genomic analyses to compare the molecular epidemiology of S14 strains globally. Our study aimed to provide a comprehensive understanding of pneumococcal infections in children, combining clinical and genomic analyses.

## Methods

### Study design and clinical data collection

This was a prospective study of children under 5-year old admitted to the fever clinic (November 1–December 31, 2019) of a territory children’s hospital in Hangzhou, China. A chest X-ray examination confirmed the diagnosis of pneumonia. The clinician determined the level of cough (0–7) and fever (0.5–7) according to the symptom severity for each patient at the time of the visit. The vaccination history of each child was obtained by inquiry during the fever clinic visit.

### Definition of febrile diseases

The final diagnosis for acute febrile illness was established based on a set of predefined clinical and microbiological criteria derived from the WHO and Infectious Diseases Society of America guidelines, as described in detail elsewhere [16]. Acute respiratory infection was defined as any acute (≤ 1 week) infection manifested by at least one respiratory sign or symptom localized to the upper or lower respiratory tract. Acute respiratory infection was further divided into two types: a child with cough and fast breathing or chest indrawing was categorized as having clinical pneumonia, and others were categorized as having upper respiratory tract infection (URTI) or bronchitis. Chest radiography findings were further used to define children with clinical pneumonia: endpoint pneumonia (alveolar consolidation and/or pleural effusion); pneumonia with other infiltrates (peribranchial thickening + / − atelectasis, compatible with the clinical entity of bronchiolitis); and pneumonia with normal chest radiography.

### Nasopharyngeal sample collection and *S. pneumoniae* isolation

Nasopharyngeal (NP) swabs were collected from each eligible child according to recommendations from the World Health Organization [17] via a Flexible minitip flocked swab (Becton, Dickinson and Company, NJ, USA) and inoculated in 1 mL of STGG medium immediately after sample collection and stored in a − 80 °C freezer until they were analyzed. Pneumococcal isolates were cultured by inoculating 100 µL of STGG medium from NP swabs onto both a 5% sheep blood agar plate (BAP) and a BAP containing gentamicin (5 µg/mL) (BIOIVD, Zhengzhou, China) and incubated under a 5% CO_2_ atmosphere at 37 °C for 18–20 h. Pneumococcus-like colonies, i.e., alpha-hemolysis and concave/mucoid morphology, were screened by optochin and bile solubility tests to identify *S. pneumoniae* strains. Finally, all pneumococcal isolates were stored in a 1.5 mL tube containing 1 mL of STGG medium at − 80 °C.

### Molecular detection of *S. pneumoniae* in nasopharyngeal specimens and density analysis

For DNA extraction, frozen NP samples were thawed at room temperature and then vortexed for 15 s. Two hundred microliters of the sample was added to 100 µl of TE buffer (10 mM Tris–HCl, 1 mM EDTA, pH 8.0) containing 0.04 g/ml lysozyme and 75 U/ml mutanolysin and then incubated for 1 h at 37 °C in a water bath. The subsequent steps were carried out according to the Qiagen DNA mini-kit protocol. The detection of *S. pneumoniae* and the analysis of bacterial density in each NP swab were performed by a quantitative PCR (qPCR) approach targeting the single copy autolysin lytA gene in all pneumococcal strains [18]. The sequences of *lytA*-qPCR primers and probes were obtained from a previous publication [19]. qPCRs were carried out in a final 10 µl volume using SsoAdvanced Universal Probes SuperMix (Bio-Rad) according to the manufacturer’s instructions. A no-template control was always included in every run. The detection limit of *lytA*-qPCR was four GEs per reaction. Negative samples were defined with cycle threshold (CT) values, if any, greater than > 40.

### Microbiology

Latex and Quellung reactions were performed for serotyping all *S. pneumoniae* isolates using pneumococcal serotype antisera (Statens Serum Institute, Copenhagen, Denmark). Molecular serotyping from whole genome sequencing (WGS) data was conducted using SeroBA (v1.0.1) [20]. Antibiotic susceptibility testing (AST) for 11 antibiotics was performed using the microdilution broth method following recommendations from the Clinical Laboratory Standards Institute, CLSI, (M100, 2019) (Supplementary Table 1). Strain ATCC 49619 was used as the quality control in all assays. Pneumococcal isolates with either an intermediate or a resistance phenotype were defined as non-susceptible (NS) strains.

### Whole-genome analysis

Genomic DNA of all *S. pneumoniae* isolates was extracted using a QIAamp DNA mini kit (Qiagen, CA, USA), and next-generation sequencing (NGS) was performed using the Illumina HiSeq2000™ platform. with a sequencing coverage of no less than 300X. Shovill [21] was used for each isolate’s paired-end fastq file assembly with a minimum length of 200 bp and minimum coverage of 10x. The assemblies were then used as input for in silico multilocus sequence typing (MLST) via PubMLST (https://pubmlst.org) and maximum likelihood phylogenetic tree building via PopPUNK [22]. The data were visualized by combining the metadata in the iTOL tree (http://ito.embl.de). The assemblies of S14 strains from other countries were downloaded from the online public database of PathogenWatch (https://pathogen.watch/), which was used for single nucleotide polymorphism (SNP) calling together with our S14 isolates via Snippy (v4.4.5) [23] to generate a global S14 phylogenetic tree that was visualized in Microreact (https://microreact.org/project/iB3KJW6gv5NYrEaf6R4pW5-allserotype14). The recombination blocks were determined in all S14 strains using a Snippy-generated alignment file in Gubbins (v2.4.1)[24] and visualized in Phandango (https://jameshadfield.github.io/phandango/#/).

### Statistical analysis

Chi-square tests were applied for risk factor comparisons; one-way ANOVA with Tukey’s post hoc test was utilized to evaluate the difference in the pneumococcal carriage density and recombination bases between serotype groups. The above statistical analyses were conducted using GraphPad Prism V9.3.1. Other than indication, the significance was determined with a *p* < 0.05.

## Results

### Clinical characteristics

Among 60 cases of respiratory infections with fever (Table 1), 56.7% (34/60) of children were male, 55% (33/60) were 37- to 60-month old, and 31.7% (19/60) lived with at least one sibling. The PCV13 and PPSV23 vaccination rates were 5.0% (3/60) and 11.7% (7/60), respectively. Severe cough and fever were identified in 23.3% (14/60) of all cases. A clinical diagnosis of respiratory infection was made in 91.6% (55/60) of all patients, of which 69.1% (38/55) had an upper respiratory tract infection (URTI), 16.3% (9/55) had bronchitis, and 14.5% (8/55) had pneumonia. *S. pneumoniae* was isolated from 50% (30/60) of all collected NP swabs. Sex (*p* = 0.0092), having siblings (*p* = 0.0125), and not being vaccinated (*p* = 0.0377) were significantly associated with an increased risk of pneumococcal colonization. A high nasopharyngeal density of Spn [> 10^4^ genome equivalent (GE)/mL] was significantly associated with severe cough and pneumonia (Table 1), and in 87.5% of pneumonia cases (7/8), a pneumococcal strain was isolated from the nasopharynx.

**Table 1:** The proportion of positive pneumococcal cases and different bacterial loads in children under 5 from the fever clinic

	All	Spn C +	C + /All	C + vs C–	Spn P +	Spn-H	Spn-L	Spn-H/P +	H vs L
	(*N* = 60)	(*N* = 30)	50.0(30/60)	*p*	(*N* = 40)	(*N* = 23)	(*N* = 17)	57.5(23/40)	*p*
Factors									
Gender									
Male	56.7(34)	73.3(22)	64.7	0.0092	65.0(26)	73.9(17)	52.9(9)	65.4	0.1692
Female	43.3(26)	26.7(8)	30.8	N/D	35.0(14)	26.1(6)	47.1(8)	42.9	N/D
Age (month)									
< 12	11.7(7)	6.7(2)	28.6	0.2276	10.0(4)	8.7(2)	11.8(2)	50.0	0.7491
12–36	33.3(20)	33.3(10)	50.0	> 0.9999	30.0(12)	39.1(9)	17.6(3)	75.0	0.1427
37–60	55(33)	60.0(18)	54.5	0.4363	60.0(24)	52.2(12)	70.6(12)	50.0	0.2399
Siblings									
0	68.3(41)	53.3(16)	39.0	N/D	62.5(25)	52.2(12)	76.5(13)	48.0	N/D
1	31.7(19)	46.7(14)	73.7	0.0125	37.5(15)	47.8(11)	23.5(4)	73.3	0.1166
Vaccination									
PCV7	0.0(0)	0.0(0)	0.0	N/D	0.0(0)	0.0(0)	0.0(0)	0.0	N/D
PCV13	5.0(3)	0.0(0)	0.0	N/D	2.5(1)	0.0(0)	5.9(1)	0.0	N/D
PPSV23	11.7(7)	6.7(2)	28.6	N/D	10(4)	4.3(1)	17.6(3)	25.0	N/D
None^a^	83.3(50)	93.3(28)	56.0	0.0377	87.5(35)	95.7(22)	76.5(13)	62.9	0.0698
Virus									
N/A	23.3(14)	16.7(5)	35.7	N/D	17.5(7)	8.7(2)	29.4(5)	28.6	N/D
Positive	31.7(19)	30.0(9)	47.4	0.7814	37.5(15)	39.1(9)	35.3(6)	60.0	0.8043
RSV	15.0(9)	20.0(6)	66.7	0.2781	20.0(8)	26.1(6)	11.8(2)	75.0	0.2629
AV	13.3(8)	6.7(2)	25.0	0.1287	12.5(5)	8.7(2)	17.6(3)	40.0	0.3974
Outcomes									
Cough									
0	23.3(14)	16.7(5)	35.7	N/D	20(8)	4.3(1)	41.2(7)	12.5	N/D
0.5–3	53.3(32)	40(12)	37.5	N/D	47.5(19)	47.8(11)	47.1(8)	57.9	N/D
4–7	23.3(14)	43.3(13)	92.9	0.0002	32.5(13)	47.8(11)	11.8(2)	84.6	0.0161
Fever									
0.5–3	76.7(46)	63.3(19)	41.3	N/D	67.5(27)	56.5(13)	82.4(14)	48.1	N/D
4–7	23.3(14)	36.7(11)	78.6	0.0146	32.5(13)	43.5(10)	17.6(3)	76.9	0.0847
Diagnose									
URTI	63.3(38)	56.7(17)	44.7	N/D	60.0(24)	47.8(11)	60(13)	45.8	N/D
Bronchitis	15.0(9)	20.0(6)	66.7	N/D	15.0(6)	21.7(5)	5.9(1)	83.3	N/D
Pneumonia	13.3(8)	23.3(7)	87.5	0.0227	17.5(7)	30.4(7)	0(0)	100.0	0.0123
Sepsis	5.0(3)	0.0(0)	0.0	N/D	2.5(1)	0(0)	5.9(1)	0.0	N/D
Flu	1.7(1)	0.0(0)	0.0	N/D	2.5(1)	0(0)	5.9(1)	0.0	N/D

^a^Three injections of PCV will be counted for an effective vaccination, two patients had only one injection for PCV13 which were counted for None

*Spn C* +  *S. pneumoniae* isolate was obtained by culture method, *C* + /*All* the proportion of *S. pneumoniae* culture positive cases in all, *C* + *vs C*– comparison between pneumococcal culture positive and negative due to different factors and outcomes, *Spn P* +  pneumococcal carriage detected by qPCR, *Spn-H* high density of pneumococcal carriage (genomic equivalent/mL ≥ 10^4^), *Spn-L* low density of pneumococcal carriage (genomic equivalent/mL < 10^4^), *H vs L* comparison between pneumococcal high and low density due to different factors and outcomes, *RSV* Respiratory Syncytial Virus, *AV* Adenovirus, *p* The *p* value of statistical significance according to the two-sided Chi-square analysis, *N/D* not determined

### Pneumococcal serotype distribution and carriage

**Fig. 1:**
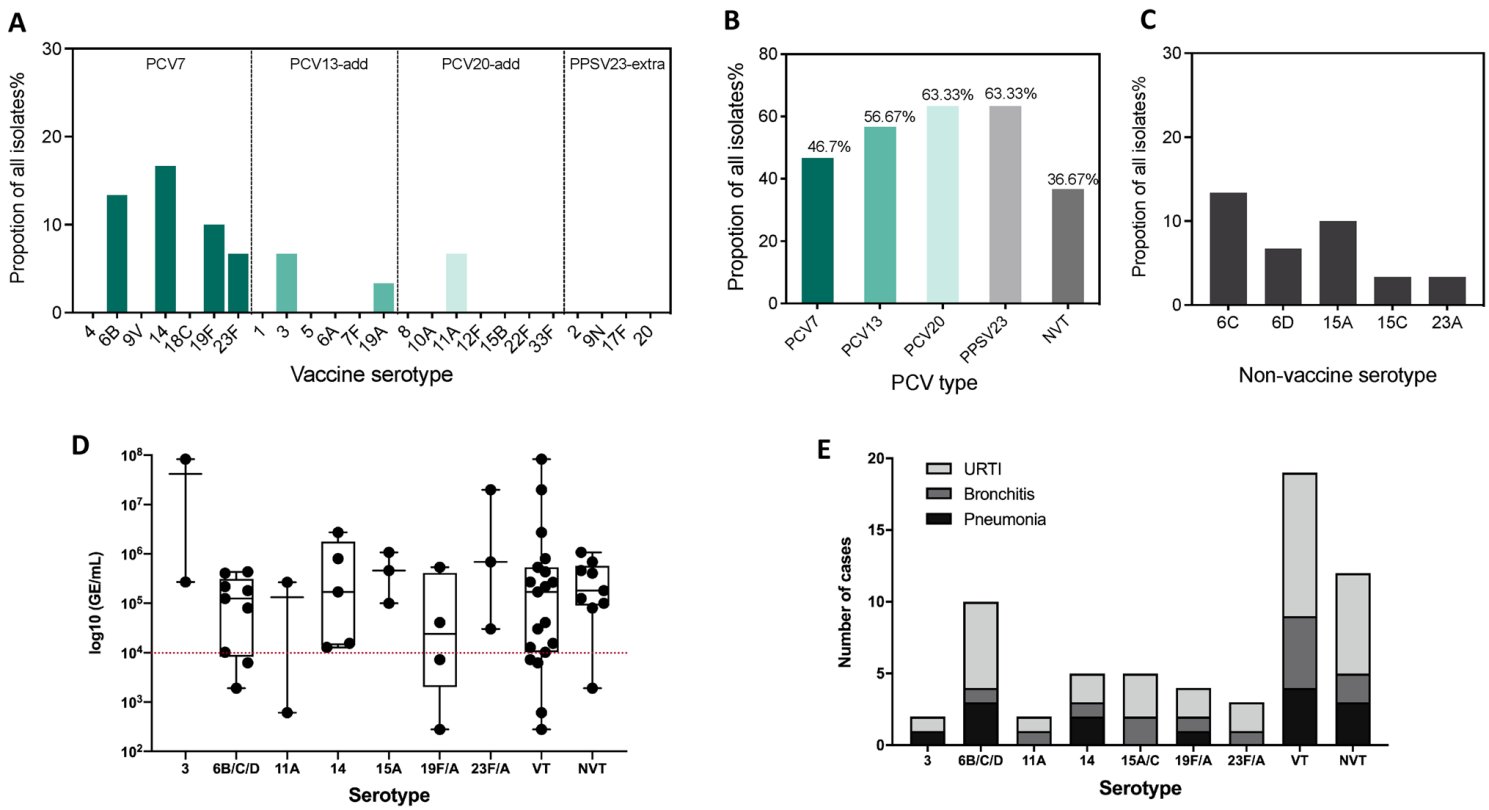
Serotype distribution and bacterial density of *Streptococcus pneumoniae* detected in nasopharyngeal specimens from children under 5. **A** The isolation rate of different pneumococcal serotypes covered by 7-valent pneumococcal conjugate vaccine (PCV7), 13-valent pneumococcal conjugate vaccine (PCV13), 20-valent pneumococcal conjugate vaccine (PCV20), and 23-valent pneumococcal polysaccharide vaccine (PPSV23); **B** the isolation rate of PCV7, PCV13, PCV20, PPSV23, and non-vaccine serotype (NVT) serotype strains. **C** The isolation rate of different NVT strains. **D** The genome equivalent (GE)/mL of different serotypes, vaccine type (VT), and NVT pneumococcal strains that were detected from nasopharyngeal samples by qRT-PCR. The red dashed line indicates a GE/ml of 10^4^. **E** Number of upper respiratory tract infection (URTI), bronchitis, and pneumonia cases that were induced by different serotypes, VT, and NVT pneumococcal strains

Serotype 14 (16.7%, 5/30), 6B (13.3%, 4/30), and 19F (10.0%, 3/30) were the most prevalent vaccine-type strains isolated in this study (Fig. 1A). PCV7 covered 46.7% of all pneumococcal isolates, while PCV13 and PCV20 serotypes were identified in 56.67% (3/30) and 63.33% (2/30) of the isolates, respectively (Fig. 1B). Non-vaccine-type (NVT) strains accounted for 36.7% (11/30) (Fig. 1C). The pneumococcal density was similar when children carrying specific serotypes were compared or whether children carried a VT compared with those carrying a NVT strain (Fig. 1D). Pneumococcal strains isolated from children diagnosed with pneumonia belonged to serotypes 3, 6B, 6C, 6D, 14, 19F, and 19A (Fig. 1E).

### Phylogenetic analysis and microbiology profile

**Fig. 2:**
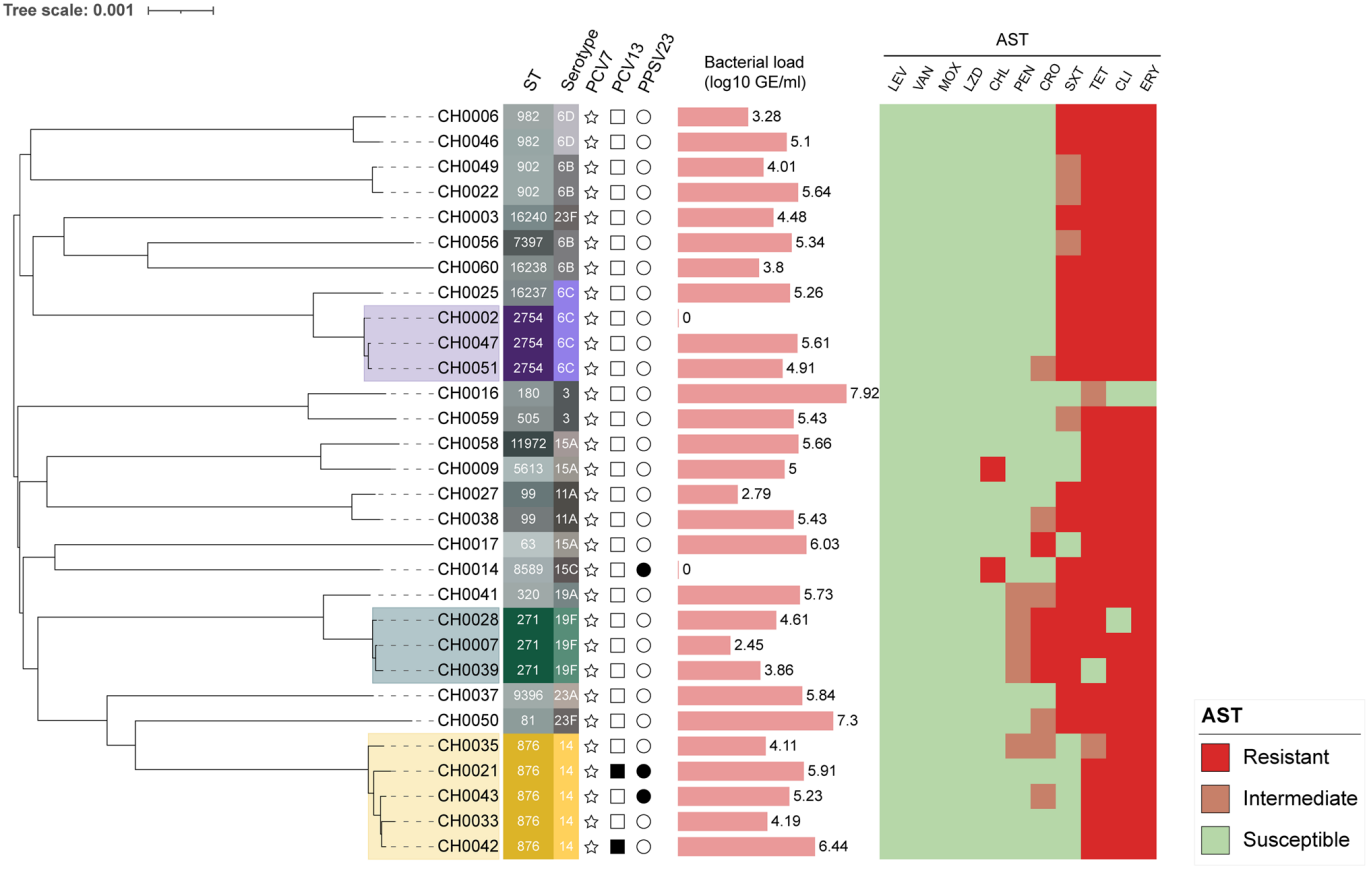
Clonal distribution, vaccine status, bacterial load, and antibiotic susceptibility of all pneumococcal isolates. The phylogenetic tree was constructed using the WGS assemblies of all pneumococcal isolates from nasopharyngeal swabs collected from children under 5 in the current study. The color strips adhering to the sample ID indicate each strain’s ST and serotype. Binary data present the vaccine status of each pneumococcal-positive case. After that, the horizontal pink bars give each pneumococcal-positive nasopharyngeal sample's bacterial load (log10 GE/mL). A heat map was drawn in the right panel according to the antibiotic susceptibility test (AST) results of all pneumococcal isolates against 11 drugs. *LEV* Levofloxacin, *VAN* Vancomycin, *MOX* Moxifloxacin, *LZD* Linezolid, *CHL* Chloromycetin, *PEN* Penicillin, *SXT* Trimethoprim/Sulfamethoxazole, *TET* Tetracycline, *CLI* Clindamycin, *ERY* Erythromycin, *CRO* Ceftriaxone

All pneumococcal strains were whole genome sequenced, and sequences were uploaded to NCBI (BioProject No. PRJNA924107). We identified 19 different sequence types (STs), of which ST876 (serotype 14), ST271 (serotype 19F), and ST2754 (serotype 6C) were the most prevalent among strains (Fig. 2). Patients who received at least one pneumococcal vaccine dose were also colonized by S14/ST876 (*n* = 3) and 15C/ST8589 (*n* = 1) strains. According to the AST results (Supplementary Table 1), all isolates were susceptible to LEV, VAN, MOX, and LZD (Fig. 2). Notably, over 90% of isolates were resistant to ERY, TET, and CLI. Most pneumococcal strains with non-susceptibility to PEN (16.7%, 5/30) and CRO (33.3%, 10/30) belonged to serotype 19F/ST271 (Fig. 2 and Supplemental Table 1).

## Global distribution of serotype 14 strains

**Fig. 3:**
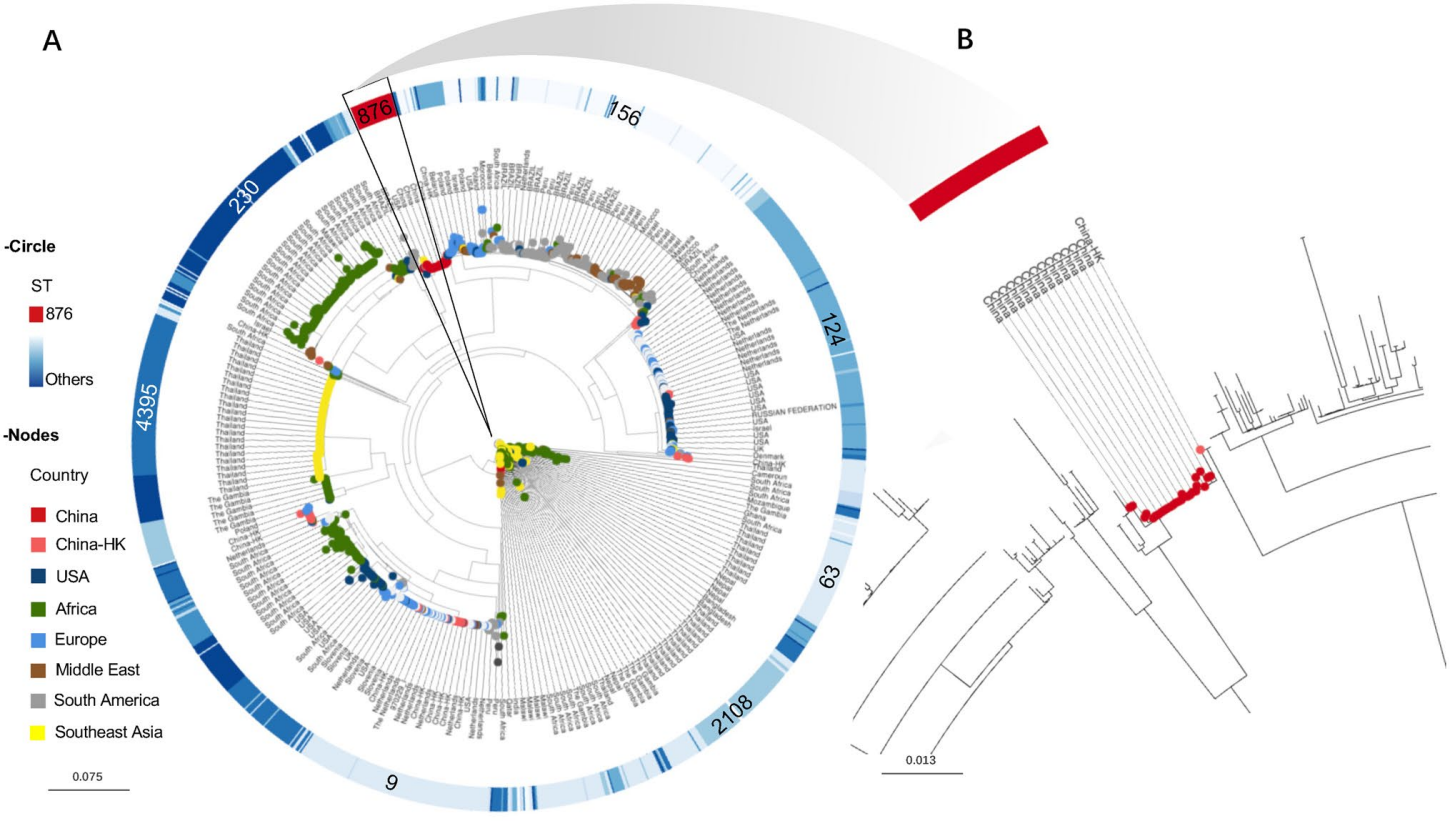
The phylogenetic structure of all *S. pneumoniae* serotype 14 strains. **A** A phylogenetic tree was constructed using PopPUNK (https://poppunk.net/) for all collected pneumococcal serotype 14 strains (*n* = 29) in our lab (11 from sputum, 8 from blood, 5 from NP, 2 from secretion, 1 from oropharynx, 1 from fester, and 1 from urine) and all serotype 14 assemblies were downloaded from the Global Pneumococcal Sequencing Project (*n* = 1468) (http://pathogen.watch). The tree was visualized via Microreact, and the data set is available at: https://microreact.org/project/6RH7SJJBCNbThyqrDXJiLL-serotype14-all. The different color strips of the circle present the STs, and the nodes aligned with branches present the country of isolation for each strain. **B** The roomed-in phylogenetic tree of pneumococcal serotype 14/ST876 strains

We then conducted a phylogenetic analysis of S14 strains (n = 1497) representing isolates from 27 countries worldwide. As shown in Fig. 3, S14/144 STs were observed, of which S14/ST63 (*n* = 234) from Africa and Southeast Asia countries was the most prevalent, followed by S14/ST156 (n = 189) from Europe and South America countries and S14/ST124 (*n* = 163) mainly from pneumococcal strains isolated in the United States and the Netherlands. Serotype 14/ST876 (*n* = 27) strains were prevalent only in China (26 from mainland China and one from Hong Kong, China). The first S14 strain was ST875, which was isolated in 1939 from a case of pneumococcal disease in Denmark. Only three S14/ST875 strain assemblies were obtained, and they were phylogenetically close to ST124 (Supplementary Fig. 1).

### Highly variant *cps* locus in serotype 14/ST876 strains

**Fig. 4:**
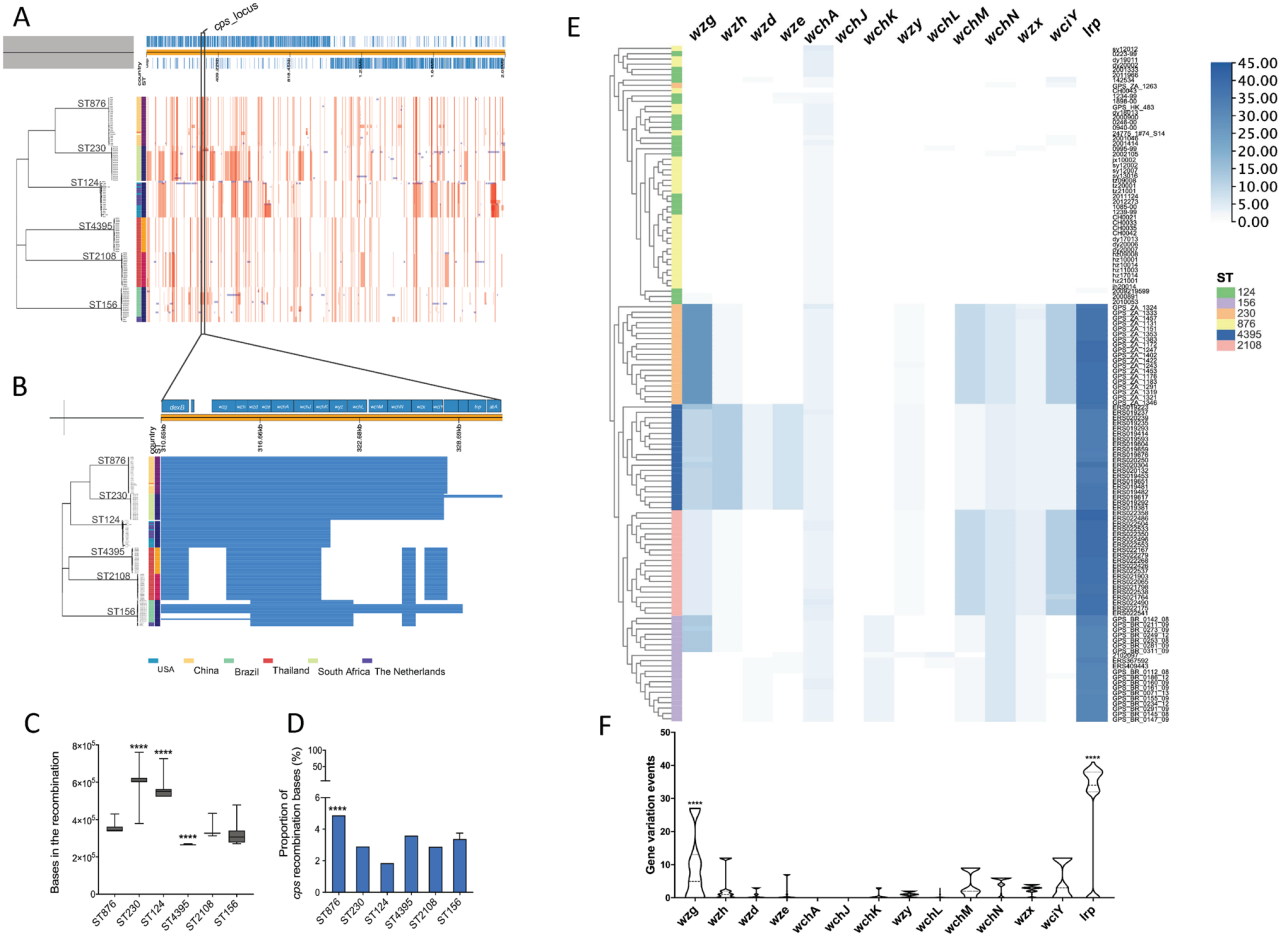
Recombination prediction and SNPs in *cps* locus of selected serotype 14 clones. **A** Recombination analysis was conducted for 6 different STs of serotype 14 strains. The red blocks present the recombination regions in all strains. **B** The magnified image of the capsule-encoding gene (*cps*) locus indicates that the recombination occurred in all *cps* locus genes for ST124 and ST876 clones. **C** The average bases (Mean ± SD) in recombination of the whole chromosome for each selected ST of serotype 14 strains. ‘****’ indicates a significant (*p* < 0.0001) difference. **D** The proportion of *cps* recombination bases in each genome for selected STs of serotype 14 strains. ‘****’ indicates a significant (*p* < 0.0001) difference. **E** A clustered tree according to the SNP numbers for each strain of all 6 selected STs of serotype 14 strains combined with a heatmap presenting the SNPs for each strain. ST876 strain CH0043 was used as the reference. (F) The average gene variation events (including SNPs, insertions, and deletions) for each gene (Mean ± SD) ‘****’ indicates a significant (*p* < 0.0001) difference

The unique prevalence of S14/ST876 strains in China prompted us to analyze their genetic variation, which was likely introduced by recombination events. Our studies revealed that the recombination blocks were distributed all over the chromosomes of selected pneumococcal strains (Fig. 4A). Notably, the *cps* locus was highly recombined in ST876 strains compared to other strains. The roomed-in image of recombination focused on the *cps* locus indicates that all pneumococcal capsule-encoding genes of the ST876 and ST230 strains were located in the region of high recombination frequency (Fig. 4B). Further analyses of genome base numbers situated in the recombination blocks indicated that ST230 and ST124 have significantly more recombined nucleic acid bases than ST876 (Fig. 4C). However, the proportion of *cps* recombined bases was the highest in the ST876 strains (Fig. 4D). Furthermore, SNP calling analysis in each gene of the *cps* locus demonstrates that gene variation of *wzg* and *lrp* is significantly different in S14/ST876 and S14/ST124 strains compared to other STs (Fig. 4EF).

## Discussion

The nasal carriage rate of *S. pneumoniae* in young children ranges from 28.5% (high-income countries) to 51% (low-income countries) [25]. As an upper-middle-income country without PCV national introduction, the reported pooled prevalence of pneumococcal nasopharyngeal carriage in China was only 21.4% and 23.8% in healthy children and children with respiratory infection symptoms, respectively [26, 27]. We demonstrated an increased carriage rate compared to what had been reported by utilizing a standardized protocol of NP sample collection and *S. pneumoniae* isolation recommended by the Centers for Disease Control and Prevention (CDC, the USA) [28]. Utilizing a standardized protocol is crucial for the epidemiological investigation of pneumococcal carriage and PD and the proper comparison of the burden within a country and worldwide [29].

An increased pneumococcal density in the nasopharynx is an important risk factor for developing pneumococcal disease [30]. Moreover, a density threshold of ~ 10^6^ GE/mL pneumococcus in the nasopharynx was associated with children’s low respiratory tract infections in other countries [31, 32]. Whereas an increased pneumococcal NP density should pose a risk of developing respiratory infections, to the best of our knowledge, no such study had been conducted in China before the current manuscript. We reported in the current study that ~ 10^4^ GE/mL was a risk factor for pneumonia in young children, suggesting that a density threshold for the development of PD might be lower in our population. Moreover, we found that pneumococcal pneumonia was significantly associated with factors such as living with siblings or being non-vaccinated, which is consistent with other studies and has special significance in the current scenario of China’s one-child policy lifting[4, 33]. Because the burden of pneumococcal disease can increase with the growth of the child population in China, our data address the importance that PCV vaccination can have for containing a future increase in PD in children and elderly individuals.

Three S14 strains were isolated from vaccinated children, the most critical strains that cause pneumococcal disease in children[34]. However, the limited understanding of the molecular epidemiology of S14 strains prompted us to study them globally. Notably, we found that S14/ST876 has only been isolated in China, whereas strains S14/ST230 and S14/ST4395 were common in South Africa and Thailand, and other STs were scattered in different countries. Some of the first strains belonging to S14/ST875 were isolated in Denmark in 1939, and whereas this particular sequence type has been prevalent in China for a long time, S14/ST875 is not the predominant S14 strain in all regions of the world except for China[35].

The dominance of S14/ST876 strains against other S14 sequence types may be due to several reasons. For instance, the misuse of beta-lactam antibiotics has perhaps served as a selection pressure for non-susceptible 14/ST876 strains, which appeared in 2001 and completely replaced ST875 in 2010 [14]. A 6-year multicenter study indicated that the increasing isolation rate of serotype 14/ST876 showed potent clonal dilation, although it was not yet a prevalent clone [12]. Our data demonstrate that non-susceptibility to beta-lactam antibiotics may not be the only reason for the potential clonal success of serotype 14/ST876, along with the increased PCV vaccination rate in China [11]. The reduced vaccine response would also be another essential factor for disseminating this clone nationally.

The nasopharyngeal carriage of S14 has been almost eradicated by the national introduction of PCVs in young children [36]. However, vaccine failure was also reported for S14, with a main driver of vaccine failure being disease caused by ST124 strains [37]. We wondered if the genetic background of different STs of S14 strains allows these strains to evade the effect of pneumococcal vaccines. Our recombination analysis indicated that S14/ST876 strains did not present the most variable genome, but indeed, these strains showed the highest recombination frequency in their *cps* locus. Moreover, the *cps* genes of S14/ST876 were almost identical to those of S14/ST124, which presented the most gene variation in the *wzg* and *lrp genes*. The gene *wzg (cpsA)* encodes an integral membrane regulatory protein, which was reported to be related to pneumococcal carriage and capsule shedding [38]. A recent study found that the yield of polysaccharides and capsule shedding in S19A pneumococcal strains was not related to mutations in *wzg,* but it was due to the difference between clones [39]. However, the function of *wzg* or mutations within *wzg* had not yet been evaluated in serotype 14 until the current study. We found that *wzg* and *lrp* (encoding a collagen-binding protein PnTIE)[40] mutations in S14/ST876 and S14/ST124 significantly differed from other STs. Hence, it is possible that the limited vaccine response in S14/ST876 strains was due to its highly variable *cps* locus and the difference in *wzg* and *lrp* genes. Further studies are needed to clarify the specific molecular mechanism by which these strains escape the effect of pneumococcal vaccines.

In conclusion, the pneumococcal carriage rate in Chinese children was underestimated mainly due to inappropriate protocols for sample collection and bacterial isolation, which is a considerable obstacle to carriage surveillance of this pathogen. PCV vaccination is now critical in China for families expecting more than one child in their household by waiving the one-child policy in China. Moreover, clone differences in *cps* locus recombination, pneumococcal capsule shedding, and binding proteins may contribute to the success of ST876 strains. This discovery provides us with a new idea for paying attention to different clone types in future research and development of vaccines.

### Supplementary Information

Below is the link to the electronic supplementary material.

Supplementary file1 (DOCX 1437 KB)

Supplementary file2 (DOCX 29 KB)

## Data Availability

All sequence data of whole-genome-sequenced pneumococcal strains were uploaded to NCBI (BioProject No. PRJNA924107). The global serotype 14 phylogenetic tree was visualized via Microreact, and the data set is available at: https://microreact.org/project/6RH7SJJBCNbThyqrDXJiLL-serotype14-all.
