# Supplementary material for: Limited protection of pneumococcal vaccines against emergent Streptococcus pneumoniae serotype 14/ST876 strains
Source: Infection. 2023 Nov 2;52(3):801–11. doi: 10.1007/s15010-023-02110-y (PMC11143005; doi:10.1007/s15010-023-02110-y)
Supplement: Supplementary file 1 — Supplementary file1 (DOCX 1437 KB) [file 15010_2023_2110_MOESM1_ESM.docx]

**Supplementary Figure legend:**

**Supplementary Figure 1. The phylogenetic structure of all *S. pneumoniae* serotype 14 strains.** A phylogenetic tree was constructed using PopPUNK (https://poppunk.net/) for all collected pneumococcal serotype 14 strains in our lab (not limited to the current study) and all serotype 14 assemblies downloaded from the Global Pneumococcal Sequencing Project (<http://pathogen.watch>). The tree was visualized via Microreact, and the data set is available at: <https://microreact.org/project/6RH7SJJBCNbThyqrDXJiLL-serotype14-all>. The different color strips of the outer, middle, and inner circle present the year of collection, the country of collection, and the sequence type (ST) for each isolate, respectively.


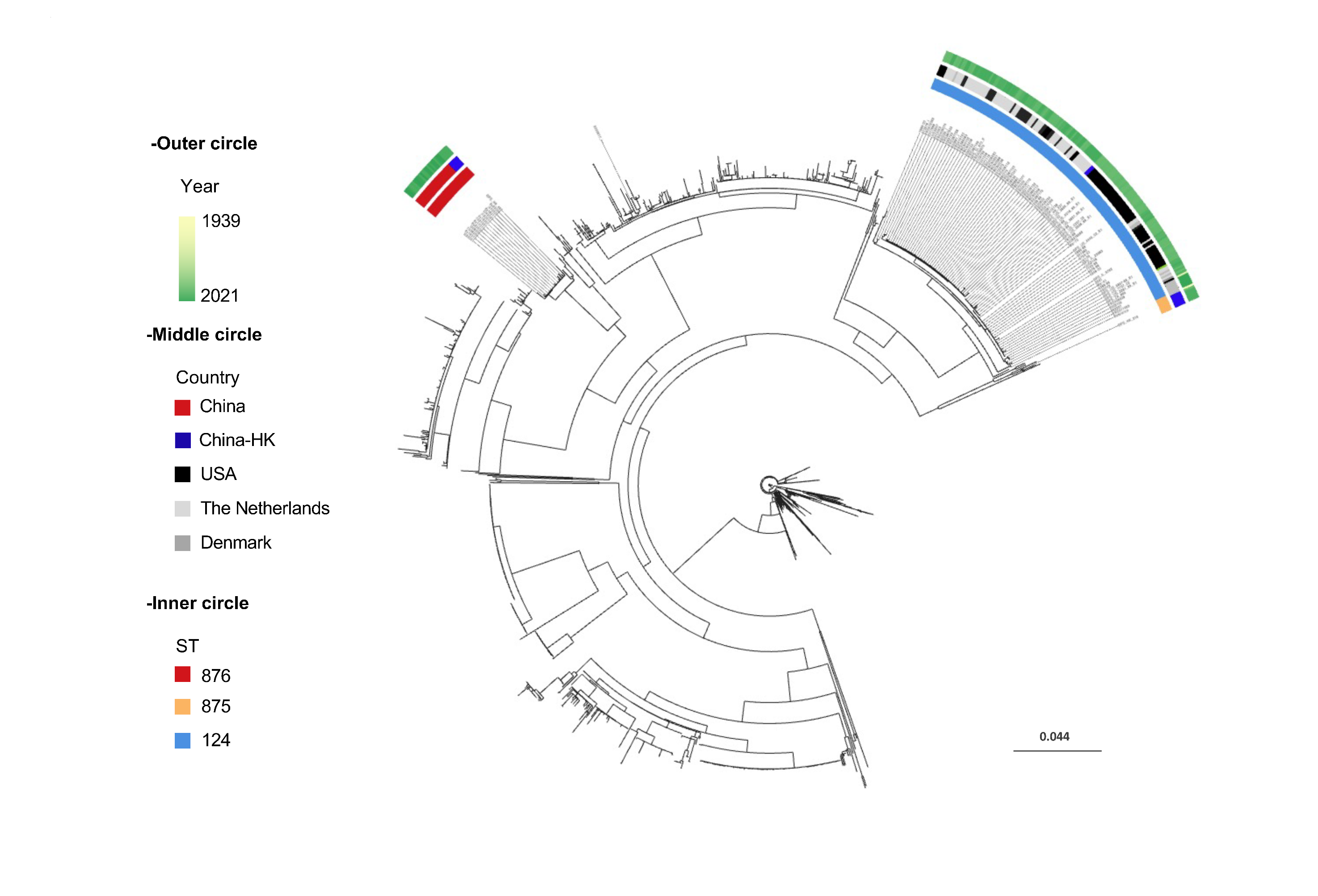
v
