# Supplementary material for: Limited protection of pneumococcal vaccines against emergent Streptococcus pneumoniae serotype 14/ST876 strains
Source: Infection. 2023 Nov 2;52(3):801–11. doi: 10.1007/s15010-023-02110-y (PMC11143005; doi:10.1007/s15010-023-02110-y)
Supplement: Supplementary file 2 — Supplementary file2 (DOCX 29 KB) [file 15010_2023_2110_MOESM2_ESM.docx]

**Supplemental Table 1.** The minimal inhibitory concentration (μg/mL) of pneumococcal isolates against tested drugs

| **ID** | **LEV** | **VAN** | **MOX** | **LZD** | **CHL** | **PEN** | **CRO** | **SXT** | **TET** | **CLI** | **ERY** |
| --- | --- | --- | --- | --- | --- | --- | --- | --- | --- | --- | --- |
| CH0002 | 0.5 | 0.25 | ≤0.5 | 1 | 4 | 0.125 | ≤0.015 | 8/152 | 64 | 128 | >128 |
| CH0003 | 1 | 0.25 | ≤0.5 | 1 | 4 | 1 | 0.125 | 4/76 | 16 | 128 | >128 |
| CH0006 | 1 | 0.25 | ≤0.5 | 1 | 4 | 0.125 | 0.06 | 8/152 | 32 | 128 | >128 |
| CH0007 | 0.5 | 0.25 | ≤0.5 | 0.5 | 2 | 4 | 4 | 8/152 | 4 | >128 | >128 |
| CH0009 | 1 | 0.25 | ≤0.5 | 1 | 8 | 0.125 | 0.03 | ≤0.5/9.5 | 8 | >128 | >128 |
| CH0014 | 1 | 0.25 | ≤0.5 | 1 | 16 | 0.125 | 0.06 | 16/304 | 64 | >128 | >128 |
| CH0016 | 1 | 0.25 | ≤0.5 | 0.5 | 1 | 1 | ≤0.015 | ≤0.5/9.5 | 2 | ≤0.06 | ≤0.06 |
| CH0017 | 0.5 | 0.25 | ≤0.5 | 1 | 4 | 2 | 4 | ≤0.5/9.5 | 32 | 128 | >128 |
| CH0021 | 1 | 0.25 | ≤0.5 | 0.5 | 2 | 2 | 1 | ≤0.5/9.5 | 8 | >128 | >128 |
| CH0022 | 0.5 | 0.25 | ≤0.5 | 1 | 4 | 1 | 1 | 1/19 | 32 | >128 | >128 |
| CH0025 | 1 | 0.25 | ≤0.5 | 1 | 4 | 0.125 | ≤0.015 | 4/76 | 32 | 128 | >128 |
| CH0027 | 1 | 0.25 | ≤0.5 | 1 | 4 | 0.125 | ≤0.015 | 4/76 | 32 | 128 | >128 |
| CH0028 | 1 | 0.25 | ≤0.5 | 0.5 | 2 | 4 | 4 | 8/152 | 8 | 0.125 | 8 |
| CH0033 | 0.5 | 0.5 | ≤0.5 | 0.5 | 2 | 2 | 1 | ≤0.5/9.5 | 4 | 128 | >128 |
| CH0035 | 1 | 0.5 | ≤0.5 | 0.5 | 2 | 4 | 2 | ≤0.5/9.5 | 2 | >128 | >128 |
| CH0037 | 1 | 0.5 | ≤0.5 | 1 | 4 | 2 | 1 | 4/76 | 16 | >128 | >128 |
| CH0038 | 1 | 0.25 | ≤0.5 | 1 | 4 | 2 | 2 | 4/76 | 32 | >128 | >128 |
| CH0039 | 1 | 0.25 | ≤0.5 | 0.5 | 2 | 4 | 4 | 8/152 | 0.25 | >128 | >128 |
| CH0041 | 1 | 0.25 | ≤0.5 | 0.5 | 2 | 4 | 2 | 4/76 | 16 | 128 | >128 |
| CH0042 | 1 | 0.25 | ≤0.5 | 0.5 | 2 | 1 | 1 | ≤0.5/9.5 | 4 | 128 | >128 |
| CH0043 | 1 | 0.25 | ≤0.5 | 0.5 | 2 | 2 | 2 | ≤0.5/9.5 | 8 | 128 | >128 |
| CH0046 | 1 | 0.25 | ≤0.5 | 1 | 4 | 0.125 | 0.03 | 8/152 | 32 | 128 | >128 |
| CH0047 | 1 | 0.25 | ≤0.5 | 1 | 2 | 0.06 | ≤0.015 | 4/76 | 32 | 128 | >128 |
| CH0049 | 1 | 0.25 | ≤0.5 | 1 | 2 | 1 | 1 | 1/19 | 32 | >128 | >128 |
| CH0050 | 1 | 0.25 | ≤0.5 | 0.5 | 2 | 2 | 2 | 4/76 | 16 | 128 | >128 |
| CH0051 | 1 | 0.25 | ≤0.5 | 1 | 2 | 2 | 2 | 4/76 | 32 | 128 | >128 |
| CH0056 | 1 | 0.25 | ≤0.5 | 0.5 | 4 | 0.25 | 0.5 | 2/38 | 16 | 128 | >128 |
| CH0058 | 1 | 0.25 | ≤0.5 | 0.5 | 4 | 2 | 1 | ≤0.5/9.5 | 4 | >128 | >128 |
| CH0059 | 1 | 0.5 | ≤0.5 | 0.5 | 2 | 0.06 | ≤0.125 | 1/19 | 32 | >128 | >128 |
| CH0060 | 0.5 | 0.25 | ≤0.5 | 0.5 | 4 | 2 | 1 | 4/76 | 32 | >128 | >128 |
| MIC_50_ | 1 | 0.25 | ≤0.5 | 0.5 | 2 | 1 | 1 | 4/76 | 16 | 128 | >128 |
| MIC_90_ | 1 | 0.5 | ≤0.5 | 1 | 4 | 4 | 4 | 8/152 | 32 | >128 | >128 |

MIC50, Minimal inhibitory concentration (MIC) inhibits 50% of isolates tested; MIC90, MIC inhibits 90% of isolates tested; LEV, Levofloxacin; VAN, Vancomycin; MOX, Moxifloxacin; LZD, Linezolid; CHL, Chloramphenicol; PEN, Penicillin; SXT, Trimethoprim/Sulfamethoxazole; TET, Tetracycline; CLI, Clindamycin; ERY, Erythromycin; CRO, Ceftriaxone.
